# Renal Pain—Modern Methods of Diagnosis and Their Interpretation

**Published:** 1917-08

**Authors:** Ernest M. Watson

**Affiliations:** 607 Electric Building, Buffalo, N. Y.


### BUFFALO MEDICAL JOURNAL

Yearly Volume 73 AUGUST, 1917 Number 1

### ORIGINAL ARTICLES

The right is reserved to decline papers not dealing with practical medical and surgical subjects, and such as might offend or fail to interest readers. Contributors are solely responsible for opinions, methods of expression and revision of proof.

### Renal Pain,—Modern Methods of Diagnosis and Their Interpretation.

ERNEST M. WATSON, A. M., M. D., Buffalo.

Renal Pain as a presenting symptom is encountered fairly frequently in most surgical lesions of the kidney and ureter. However, not all pain in the region of the kidney has its origin along the urinary tract and with this symptom disease of the appendix, gall bladder, hepatic and splenic flexures of the colon, retro-peritoneal growths and an occasional psoas abscess have to be borne in mind. Along the urinary tract it is possible to localize lesions with great accuracy and at the present time its elimination as a source of trouble is most desirable in all cases where the exact diagnosis is in doubt. Modern methods of examination with functional studies have made it possible to consider this system in situ with almost as much knowledge as if we had it removed from the body and in a specimen jar.

(1). The routine of a kidney study should begin with a careful examination of the bladder urine in a centrifuged specimen from the third glass of the voided specimens. The determination of the presence or absence of albumen, pus, blood and infection are essential for the further intelligent interpretation of the findings.

(2). A plain X-ray of the kidneys, ureters and bladder should be the next step in the study of these cases. If these are negative or if suspicious shadows are seen and the diag- should be taken making stereoscopic plates. Small faint shadows often overlooked or considered unimportant in the nosis has not been helped by this procedure a second series plain negatives will often assume a much greater significance when seen in the stereoscopic plates.

(3). After the X-rays a functional kidney test should be made preferably with phenol sulphone phthalein. This should be a total functional test of both kidneys. The dye should be injected intramuscularly and the specimens collected by voiding or by catheterization if there is residual urine present or if the patient is unable to void, at the end of an hour and ten minutes and two hours and ten minutes respectively. This will determine the total working capacity of both kidneys.

(4). Following this a cystoscopy and a ureteral catheterization is in order to determine the character of the urine and the functional capacity of each kidney separately. In this regard the color of the urine, the amount, the microscopic elements, with a study for infection and the urea content are necessary. The phthalein test for the determination of divided function should be given intra-venously the appearance time noted, the amount of urine excreted in a half hour and the amount of phthalein excreted. While the total phthalein may be normal due to a compensatory activity of the well kidney, the divided function in unilateral disease will reveal a reduced output on the affected side.

(5). If the foregoing studies are negative or if there is need of additional information pyelograms should be made of the renal pelvis and the plate should be made also to include the ureter on the suspected side. These are best made after an injection by gravity of a 15% solution of thorium as recommended by Burns (1). By this means anomalies, anatonical variations and even small calculi which fail to appear in the plain plates may be localized with surprising accuracy.

(6). A further procedure, the importance of which can be appreciated only after its demonstration in certain puzzling cases, is the passage of the wax-tipped catheter. By this means of employing the method suggested by Kelly (2) and later amplified by Burton Harris (3), Hinman (4) and Kirkendall (5) may be located small ureteral calculi too small to cast a shadow on X-ray examination. No case of hematuria, unilateral hydronephrosis or renal colic in which the cause is uncertain can be considered thoroughly worked up until one has the data revealed by the passage of the wax-tipped catheter. Oftentimes also a persistent unilateral pyelitis may be kept up by the presence of a small calculus partially obstructing the ureter but giving no other symptoms and failing to show on the X-ray plates.

Of the lesions in the upper urinary tract calculi occupy an extremely important place. These calculi may be in the parenchyma of the kidney, in the calyces, pelvis, along the ureter or they may have passed and be found in the bladder. Small calculi in the renal parenchyma, as a rule, cause fewer symptoms than those at any other site and often no symptoms are given. A parenchyma calculus can be diagnosed by only one means and that is the plain X-ray of the kidney. In the pelvis and calyces, however, calculi are wont to give considerable pain and frequently hematuria. Their presence there may be detected by the plain X-ray or if this is negative or only suspicious shadows are seen their density may be increased by injecting into the renal pelvis a 15% solution of thorium through a ureteral catheter and then allowing it to drain off. The calculi will absorb in this way a small amount of the thorium solution and upon subsequent X-ray examination their shadow will be intensified and outline clearly delineated. The location of election for ureteral calculi are for the most part confined to the three sites of anatomical narrowing of the ureter. These are first about the uretero-pelvic junction, second in the region of the pelvic portion where the ureter bends over the superior strait into the pelvis proper, and third about the junction of the ureter and bladder or in the intramural portion of the ureter. The diagnosis of calculus in the ureter may be accomplished by plain X-ray, and if this is negative by X-rays taken after the ureter has been injected with a solution of thorium to intensify the shadow. If these two procedures are negative the passage of a wax-tipped catheter is practically certain of establishing a diagnosis. In Geraghty's (6) experience there has been only one instance in which the wax-tip method failed to reveal the calculus when present. In this case there was a sacculation of the ureter which held the calculus and allowed the wax-tip to pass by without receiving any scratch. In renal colic with a suggestive history of ureteral calculi the bladder also should be examined cystoscopically with considerable care for not infrequently the calculi pass spontaneously and may occasionally be found in the bladder.

Another group of kidney lesions in which the pain may be severe, at times constant at others intermittent in character and not uncommonly giving a real renal colic are those of infections (non-tubercular). In this class may be considered pyelitis, pyelo-nephritis and pyo-nephrosis. The simple pyelitis may readily be diagnosed by obtaining pus and organisms from the affected side through the ureteral catheter and in the functional studies finding no reduction in the output of phthalein. The border line between a simple pyelitis and a pyelo-nephritis is not always easy to determine but a good working basis may be taken that when we begin to find a definite reduction in the phthalein excretion we are safe in assuming that there has been a destruction of kidney substance. If the phthalein output begins to fall much below 20% for a half hour from one kidney after an intra-venous injection,—this finding with pus and organisms means a definite pyelo-nephritis. When the function falls still lower or is merely a trace or nothing in a half hour and there are large amounts of pus and bacteria present and when often times a spurt of creamy fluid can be seen through the cystoscope escaping from the ureter on the affected side before the introduction of the ureteral catheter we know that we are dealing with a frank pyo-nephrosis or pus kidney.

In tuberculous infections of the kidney it is rare that an individual consults a urologist early enough for him to diagnose a tuberculous pyelitis though such does happen occasionally. More often the condition has advanced to a pyelo-nephritis and frequently to a pyo-nephrosis. The diagnosis here is confirmed by the finding of the tubercle bacilli from the affected side and also in the bladder urine. Usually by the ordinary methods of staining no organisms but considerable quantities of pus are found and the demonstration of the acid-fast organisms is dependent in the vast majority of cases upon the diligence of the examiner in searching for them.

In the so-called idiopathic or essential hematuria the diagnosis is determined solely by a process of elimination. With negative pyelograms and plain X-rays and no scratches revealed after passing the wax-tipped catheter the diagnosis is practically assured. In addition with no infection present and a normal function there can be no other possibility for the presence of the hematuria except a papilloma in the pelvis of the kidney of which there are only a few reported cases in urological literature.

Tumors of the kidney, of which the most common is the hypernaphroma, are diagnosed with difficulty except when they reach a size that is readily palpable. The X-ray findings may help in outlining a mass if it is of appreciable size and density. The pyelograms and urinary findings are not characteristic. The functional studies beyond showing a reduction due to the destruction of renal tissue are of little avail and the diagnosis in the early cases must be made by exclusion of other conditions and with the clinical history and picture.

Of the anomalies of the kidney and ureter that give pain as the most characteristic symptom may be mentioned hydronephrosis, horse-shoe kidney, movable kidney, kink in the ureter, double ureter and reduplicated pelvis, and the so-called "Fixed ureter." Hydronephrosis is a condition easily and absolutely diagnosed by pyelograms. In a unilateral dilated pelvis one should always examine the ureter on the affected side for evidences of obstruction, namely a stricture, kink or a small calculus. Oftentimes it is possible to save such a kidney if we remove the obstruction below and do a Mikulicz plastic operation at the ureto-pelvic junction. The passage of a ureteral bougie and a wax-tipped catheter will always rule out these conditions as etiological factors. The horse-shoe kidney (7) will also be readily revealed on examination of the pyelographic plates. Movable kidney is a condition not infrequently met with and occasionally escapes recognition even after repeated examination. It can be diagnosed definitely after two sets of pyelograms are taken one with the patient in the upright position and another with the patient lying flat on the back or with the head and trunk lowered. The same procedure should be followed in locating a kink in the ureter. In this instance it is well to insert the ureteral catheters only a few centimeters up the ureter so that the natural form and outline of the ureter may be observed. A ureteral catheter inserted clear to the pelvis may occasionally straighten out a very disturbing kink in the ureter.

Pain in the kidney region and even severe renal colic with negative X-rays, normal urine and normal kidney function and negative wax-tip examination is occasionally due to a reduplicated ureter and pelvis. This is found on taking a pyelogram in which the condition is clearly and strikingly demonstrated.

Another condition which seems to be a very definite lesion in the etiology of renal colic is a "fixed ureter," or one bound down by abnormal adhesions. Examination in this instance is absolutely negative throughout. The pyelograms particularly show absolutely no deviation from the normal. This condition occurs in young men and is apparently without known cause. This is the type of case recently mentioned by Pilcher (8). During the past two years three cases of this type occurred in the service of Dr. J. T. Geraghty at the Brady Urological Institute and the writer who had the privilege of caring for these patients on the wards can speak positively of the relief of symptoms accorded these individuals by a simple freeing of the ureter from the kidney pelvis to about half way above the pelvic brim.

The symptom of renal pain and also a typical colic is perhaps always due to a definite lesion. Fortunately though practically all such lesions lend themselves to treatment with highly satisfactory results. The important element in the management of these cases is to thoroughly understand the cause of the symptoms. This knowledge at the present time is readily obtainable by the modern diagnostic methods and in all instances where uncertainty exists a thorough renal study is not only indicated but will invariably yield the best results for both patient and physician.

607 Electric Building, Buffalo, N. Y.

BIBLIOGRAPHY.

(1). Burns. Thorium—A new Agent for Pyelography: Jour. A. M. A. Vol. LXIV. p. 2126, June 26, 1915.

(2). Kelly. The Diagnosis of Renal Calculus in Women. Med. News. Vol. LXVII. p. 593, 1895.

(3). Harris. The Diagnosis of Ureteral Calculus by Means of the wax-tipped whale bone bougie used with the Nitze Cystoscope. Surg. Gyn. & Obstet. Vol. XV. p. 727, 1912.

(4). Hinman. A Practical Method of Applying the Wax-tipped Catheter in the Diagnosis of Ureteral Stone in the Male. Jour. A. M. A. Vol. LXIV. p. 2129, June 26, 1915.

(5). Kirkendall. I. A New Method of Passing the Wax Tip. II. Modification of the Technic of Passing the Wax Tip. Jour. A. M. A. Vol. LXV. p. 1253, Oct. 9, 1915.

(6). Geraghty. Verbal Communication.

(7). Burns. Thorium—A New Agent for Pyelography. The Johns Hopkins Hospital Bulletin. Vol. XXVII. No. 304, p. 157. 1916.

(8). Pilcher. Pain due to Anatomical Variations of the Ureter. Long Island Med. Jour. Vol. XI. p. 1, 1917.

---

**Non-Specificity of Tuberculin Reactions.** Fishberg, Pulm. Tuberculosis, book, pages 32 and 319, considers the various reactions due to supersensitiveness to foreign proteins generally in harmony with Victor C. Vaughan's theory of protein intoxication. Thus tuberculous individuals may react positively to various other bacterial toxins while persons not actually infected with tuberculosis, may react positively to many of the tuberculin tests. However, the tests are not without value for generally speaking, a supersensitive individual is liable to infection.

